# Impact of climate change on potential distribution of *Dickeya zeae* causal agent of stalk rot of maize in Sialkot district Pakistan

**DOI:** 10.1038/s41598-024-52668-2

**Published:** 2024-01-31

**Authors:** Humaira Shahid, Sajjad Hyder, Muhammad Naeem, Anam Sehar, Amjad Shahzad Gondal, Zarrin Fatima Rizvi, Rashid Iqbal, Muhammed Habib ur Rahman, Mona S. Alwahibi, Mohamed S. Elshikh, Muhammad Ayaz, Muhammad Arslan, Sergio de los Santos-Villalobos, Amelia C. Montoya-Martínez

**Affiliations:** 1 Department of Botany, Government College Women University Sialkot, Sialkot, 51310 Pakistan; 2 Department of Zoology, Riphah International University Faisalabad Campus, Faisalabad, 44000 Pakistan; 3 Department of Student Affairs and Counselling, Lahore Garrison University, Lahore, 54000 Pakistan; 4 Department of Plant Pathology, Bahauddin Zakariya University, Multan, 60000 Pakistan; 5 Department of Agronomy, Faculty of Agriculture and Environment, The Islamia University of Bahawalpur, Bahawapur, 63100 Pakistan; 6 Institute of Crop Science and Resource Conservation (INRES), University of Bonn, 53115 Bonn, Germany; 7 Department of Seed Science and Technology, Institute of Plant Breeding and Biotechnology (IPBB), MNS-University of Agriculture, Multan, 66000 Pakistan; 8 Department of Botany and Microbiology, College of Science, King Saud University, 11451 Riyadh, Saudi Arabia; 9 Lithuanian Research Centre for Agriculture and Forestry, Institute of Agriculture, Instituto Al. 1, 58344 Akademija, Kėdainiai Dist., Lithuania; 10 Vytautas Magnus University Agriculture Academy Bioeconomy Research Institute, Studentų Str. 11, 53361 Akademija, Kauno R., Lithuania; 11 Institute of Agroecology and Organic Farming Group, University of Bonn, 53115 Bonn, Germany; 12 Departamento de Ciencias Agronomicas y Veterinarias, Instituto Tecnologico de Sonora, 85010 Ciudad Obregon, SO Mexico

**Keywords:** Biochemistry, Developmental biology, Immunology

## Abstract

Maize (*Zea mays*) is an influential crop in its production across the world. However, the invasion of many phytopathogens greatly affects the maize crop yield at various hotspot areas. Of many diseases, bacterial stalk rot of maize caused by *Dickeya zeae* results in severe yield reduction, thus the need for efficient management is important. Further, to produce epidemiological information for control of disease outbreaks in the hot spot regions of Sialkot District, Punjab Pakistan, extensive field surveys during 2021 showed that out of 266 visited areas, the highest disease incidence ranging from 66.5 to 78.5% while the lowest incidence was ranging from 9 to 20%. The Maxent modeling revealed that among 19 environmental variables, four variables including temperature seasonality (bio-4), mean temperature of the wettest quarter (bio-8), annual precipitation (bio-12), and precipitation of driest month (bio-14) were significantly contributing to disease distribution in current and coming years. The study outcomes revealed that disease spread will likely increase across four tehsils of Sialkot over the years 2050 and 2070. Our findings will be helpful to policymakers and researchers in devising effective disease management strategies against bacterial stalk rot of maize outbreaks in Sialkot, Pakistan.

## Introduction

*Zea mays* belongs to the Poaceae family^1^ and it is the 3rd most important crop by its production across the world, where rapidly increasing human population has already out-stripped the available food supplies. It plays a major role in food security in many developing countries in Asia and Africa. In Pakistan, maize is classified as the 4th most influential crop after wheat, rice, and cotton and its yield has increased by 6% during 2019–2020 which contributes 0.6% to the gross domestic production of Pakistan^2^. The 97% volume of the total maize production is achieved from two major provinces of Pakistan where Khyber Pakhtunkhwa (KPK) contributes 57% area and produces 68% of the total yield while in Punjab, maize covers 38% area and shares 30% of total maize yield. On the other side, Sindh and Balochistan provinces contribute only 2–3% of the total maize production^3^.

In Pakistan, 65% of maize is cultivated on irrigated lands while the rest is grown on dryland^4^. Maize, being a famous kharif crop (monsoon season crop), is also widely used as forage for domestic animals as well as for poultry^5^. Maize grains serve as a rich source of vitamins A, and B3, starch, oil, proteins, sugar, fiber, carbohydrates, and ash^6^. Apart from this, it serves as a raw material to produce corn oil, dextrose, corn syrup, corn flakes, wax, and cosmetics^7^, ethanol production^8^, and serves as a major source of calories in animal ingestion and feed sensationalism^9^.

Maize has a high economic value after rice and wheat and is grown at a large scale due to its ability to survive under various climatic conditions^10^. Pakistan offers the right set of conditions to enhance maize production, but many limitations impart bad effects on maize production. Many living and non-living factors result in yield losses in maize crops. Among various biotic factors, bacterial stalk rot of maize (BSRM) caused by *Dickeya zeae* (syn. *Erwinia chrysanthemi* pv. *zeae*) is of major concern and causes significant yield losses in maize, adversely affects the quality of the produce if left untreated^11,12^. Infected stalks produce an unpleasant smell and topple down of plants causing acute yield losses of up to 98.8%^13^.

Infection caused by *D. zeae* on maize becomes severe with the change in climatic conditions, and disease incidence increases with the increase in temperature and high humidity^14,15^. *Dickeya zeae* has a wide host range and spreads through rainwater from individual plants to the whole maize field^16,17^. Both high temperature and relative humidity favour the physiological and metabolic activities of the pathogen due to which bacterium grow well and produce sufficient pectolytic enzymes which degrade the plant cell^13^.

Geographic Information Systems (GIS) together with species distribution modeling (SDM) approaches have already been used in studying species and/or disease distribution as well as their forecasting^18–22^. Both GIS and SDM are influential tools for a better understanding of spatial disease distribution^23^. Also, the GIS facilitates the growers to take appropriate action well in time before the disease outbreak^24^. This technology is widely adopted as an important tool in epidemiological study of plant diseases. Wetwood disease is one of the most influential diseases on elm trees observed in the Northwest of Iran in Tabriz city and become terribly epidemic. The epidemiological assessment of wetwood disease on elm trees was studied by using geographic information system GIS^18^.

The proposed study aimed at estimating the stalk rot disease incidence, and severity on maize crops cultivated in the Sialkot district, Pakistan. Data collected on the stalk rot of maize and different bioclimatic layers were used in disease predictive modeling and forecasting. Maxent modeling was employed to predict regions with a high chance of disease spread based on disease occurrence and bioclimatic data. This model was further used to forecast the disease spread in coming years^25,26^. Predictive modeling, high risk of potential distribution, and high chances of BSRM in new areas could help design and adopt the disease management strategies in advance to cope with the potential yield loss due to bacterial stalk rot disease in maize crops. This study is helpful to the farmer community (growers) to understand the stalk rot of maize disease occurrence trends in the coming years and help them devise preventive disease management strategies before the disease outbreak. As no data on the potential distribution of BSRM is available in Pakistan, the current research was carried out to understand the potential distribution of *D. zeae* in maize-growing areas and predictive modeling for the disease outbreak in the coming years. To our knowledge, this is the first research study on this stated subject from Pakistan.

## Materials and methods

### Description of the locations of study

**Figure 1:**
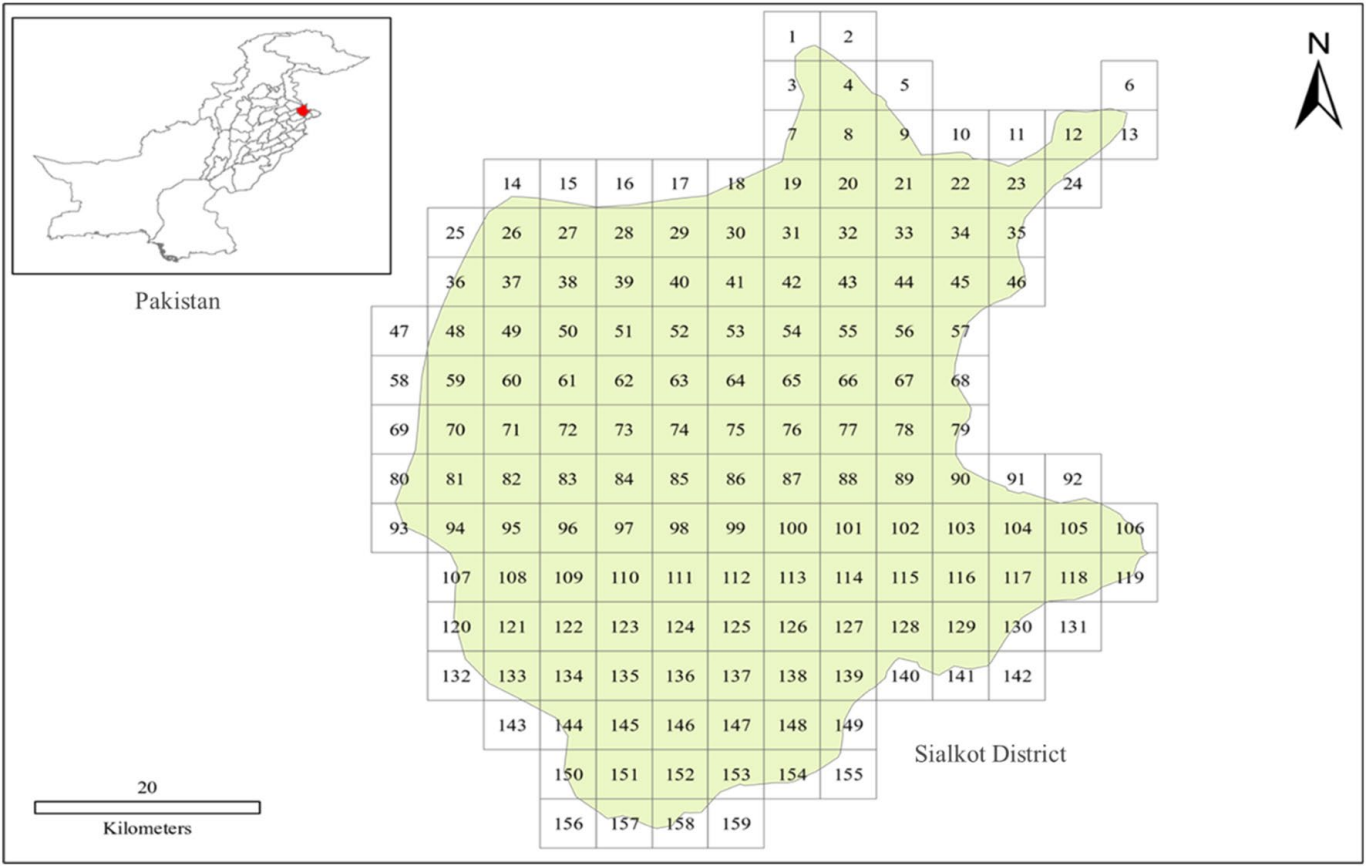
Map of Sialkot District, Punjab Pakistan. The area in Sialkot District was divided into a total number of 159 small grid cells each covering an area of 5 × 5 km^2^.

The rural areas in the Sialkot district of Punjab, Pakistan were selected for this research study. It is situated at the foot of Kashmir hills, near the river Chenab, in the northeast of Punjab, Pakistan (Fig. 1). Sialkot is bounded on the northeast by the Jammu and Kashmir state, on the north-west by two rivers (Ravi and Chenab) which separate it from the Gujrat, on the west and southeast by Gujranwala and Narowal districts respectively. Sialkot is located at 32.4945° N, 74.5229° E with an elevation of about 840 ft above sea level.

The average monthly temperature in Sialkot ranges from 11.6 °C in January to 32.2 °C in June^27^. Monsoon is the source of precipitation in this region, and the mean annual rainfall is 957 mm, over half of which falls in the summer monsoon which often results in flooding^28^. The study area was divided into 159 grid cells of 5 × 5 km^2^ (Fig. 1). The major cultivated crops in the district of Sialkot are rice, wheat, and maize^29^. Kharif (monsoon) season is the main growing season of maize crop. However, it may be planted at any time from March to October.

### Field survey for bacterial stalk rot of maize disease assessment

A field survey to assess the occurrence of BSRM was conducted from May to July 2021 in Sialkot district, Pakistan. All four tehsils in district Sialkot including Daska, Pasrur, Sambriyal, and Sialkot were systematically inspected. From each of the selected sites, five fields from every 5 km^2^ distance were visited. From each field, five points (5 m × 5 m area) were randomly inspected, and maize plants were observed for disease assessment. Field coordinates from each visited location were recorded by using GPS Essentials ArcGIS 10.4 v and MaxEnt v. 3.3 software. Names of the visited areas in Sialkot district along with the GPS coordinates in Degrees, Minutes, and Seconds (DMS) notation are presented as supplementary material.

Disease incidence (*D*_*I*_) and severity (*D*_*s*_)^30^ were recorded from each visited location according to the given formulas reported by Tahir et al.^30^ Disease incidence percentage (*D*_*IP*_) was calculated by using the formula given below:

$$DIP{{ \% }} = { }\frac{{{\text{Total}}\;{\text{ No}}.\;{\text{ of }}\;{\text{Infected }}\;{\text{Plants}}}}{{{\text{Total }}\;{\text{No}}.\;{\text{ of}}\;{\text{ Observed }}\;{\text{Plants}}}}{ }\; \times { }\;100{ }$$

Disease severity was recorded by using the disease severity rating scale^31^, and the disease severity percentage (*D*_*SP*_) was calculated using the following formula:

$$DSP{{ \% }} = { }\frac{{{\text{Some}}\;{\text{ of}}\;{\text{ the}}\;{\text{ Rating}}}}{{{\text{Total}}\;{\text{ No}}.\;{\text{ of}}\;{\text{ plants}}\;{\text{ observed}}\; \times {\text{Rating}}\;{\text{ scale}}}}{ } \times { }100{ }$$

### Isolation of *Dickeya zeae* from infected plant samples

Maize stalks showing the typical browning and necrosis symptoms were sampled for the isolation of bacteria. Infected segments were surface disinfected in ethanol (70%) for 5 min and 1% NaClO followed by five consecutive washings in sterilized distilled water^32^. Disinfected plant parts were aseptically crushed in distilled water and streaked on nutrient agar (NA) containing Petri plates and incubated at 26 ± 2 °C for 48h^15^. After incubation, morphological discrete bacterial colonies were picked aseptically and cultured on freshly prepared NGM medium (23 g of NA, 10 ml of glycerol (1% v/v), and 0.4 g of 2 mM MnCl_2_.4H_2_O/1000 ml) carrying Petri plates for detecting the production of characteristic pigments of *D. zeae*^33^. All the bacterial strains were preserved at − 80 °C in a solution of NB and 80% glycerol.

### Biochemical characterization of *Dickeya zeae*

The biochemical characterization of *D. zeae* was carried out following previously reported methodologies: For catalase activity^34^, a 3% H_2_O_2_ on a glass slide, to show the formation of gas bubbles was performed according to Hayward et al.^34^ The procedure of Dasri et al.^35^ was followed to test bacterial motility and indole-3-acetic acid production^35^. Hydrogen sulfide production^36^ was tested following the methodology of Warren et al. A nutrient gelatin stab method was used for the determination of gelatin liquefication^37^. Nitrate reduction was assessed with Griess Llosvay reagents^38^ following the protocol of Choi, et al.^38^ Starch hydrolysis^39^, urease activity^40^, and levan production^41^ were also tested, for the characterizations of the *D. zeae* isolates.

### Bioclimatic/Environmental variables

A total of 19 bioclimatic layers were obtained from the WorldClim database ver. 1.4 (www. worldclim.org)^42^. These layers are classified into two categories including temperature variables and precipitation which are presented in Table 1. Future climatic data for the years 2050 and 2070 was downloaded from CliMond^43^ based on the global climate model, CSIRO-Mk3.0 with the Intergovernmental Panel on Climate Change^44^.

**Table 1:** Bioclimatic layers used for predictive modeling of bacterial stalk rot maize disease by Maxent Software^45^.

Variable	Description	Unit
bio1	Annual mean temperature	°C
bio2	Mean Diurnal Range (Mean of Monthly (max temp-min temp)	°C
bio3	Isothermality (bio2/bio7) (*100)	–
bio4	Temperature seasonality (standard deviation *100)	°C
bio5	Max temperature of the warmest month	°C
bio6	Min temperature of the coldest month	°C
bio7	Temperature Annual Range (BIO5–BIO6)	°C
bio8	Mean temperature of wettest quarter	°C
bio9	Mean temperature of driest quarter	°C
bio10	Mean temperature of warmest quarter	°C
bio11	Mean temperature of coldest quarter	°C
bio12	Annual precipitation	mm
bio13	Precipitation of the wettest month	mm
bio14	Precipitation of the driest month	mm
bio15	Precipitation seasonality (Coefficient of variation)	mm
bio16	Precipitation of the wettest quarter	mm
bio17	Precipitation of the driest quarter	mm
bio18	Precipitation of the warmest quarter	mm
bio19	Precipitation of the coldest quarter	mm

### Modeling procedure and GIS analyses

A pairwise Pearson correlation analysis was performed to overcome the multicollinearity of the bioclimatic/environmental variables by using ENMTools^46^. Based on percentage contribution, variables with a lower than 0.8 value were kept for model fitting. However, in the case of variables higher than 0.8 and highest contribution was selected^45^. The spatial resolution of bioclimatic variables was at 30 arc-seconds (~ 1 km). All these variables were clipped to match the dimensions of district Sialkot, Punjab Pakistan, and saved in ASCII grid format for further use in the MaxEnt program. ArcGIS software v. 10 was used for clipping these bioclimatic layers. The distributions of *D. zeae* were produced by using MaxEnt software ver.3.3. This software is most influential because it produces and records useful predictions related to species distribution in study area^20^. All the variables were converted to ASCII files in ArcToolbox 2.0 in ArcGIS^47^. Data related to stalk rot of maize distribution was also saved in comma-separated value format in an Excel program. The predicted distribution of stalk rot of maize was analyzed and reclassified into different groups. Moreover, the area of distribution was estimated in square kilometers with a zonal statistical analysis program in ArcGIS software.

### Model validation and potential habitat prediction

Of all the methods, an area under the curve (AUC) is the most suitable method for evaluating the model accuracy^48,49^. Jackknife test was performed using the MaxEnt software to determine the predictive performances of all the selected variables by the adopted method from Pearson et al.^50^. The response curves were generated to observe how each bioclimatic layer affects the Maxent prediction, and how the logistic prediction varies under varying bioclimatic variables^48^. The values for AUC were theoretically set between 0.5 and 1. AUC value closer to 1.0 indicates a successful model with clear distinction while an AUC value closer to 0.5 reflects a model with no clear distinction^51^. The model classification index used in this study was: “failure” is 0.50 < AUC < 0.60, “poor” is 0.60 < AUC < 0.70, “fair” is 0.70 < AUC < 0.80, “good” is 0.80 < AUC < 0.90, and “excellent” is 0.90 < AUC < 1.00^52,53^. Both the curves, such as ROC and AUC were employed for the evaluation of accuracy of the disease distribution model^54^.

### Spatial conservation assessment

The models of current and future distribution were compared with the surveyed areas of BSRM disease in 2021. The accuracy and fitting of models between different periods and all the surveyed areas were also analyzed. The current distribution of stalk rot of maize with future potential distributions in coming years i.e., 2050 and 2070 were combined. For calculating the suitable overlapping areas, the raster calculator was used according to the recently reported methods^21,55^.

### Ethics approval and consent to participate

This study does not include human or animal subjects.

### Statement on guidelines

All experimental studies and experimental materials involved in this research are in full compliance with relevant institutional, national and international guidelines and legislation.

## Results

### Bacterial stalk rot of maize disease assessment survey

**Figure 2:**
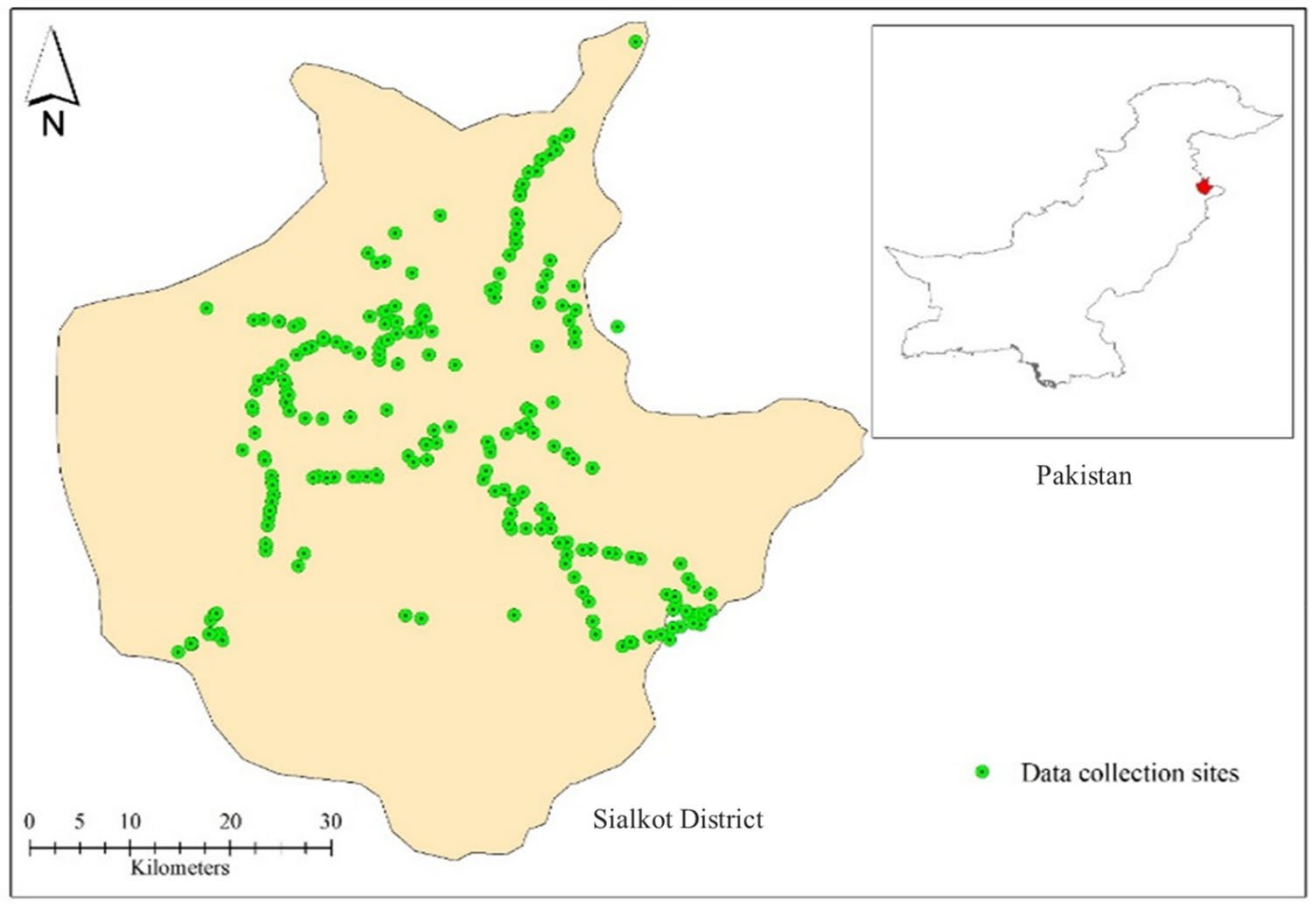
Pictorial representation of the data collection sites in district Sialkot, Pakistan.

All the tehsils in district Sialkot were inspected for BSRM disease assessment recording. The disease data and sampling sites are presented in Fig. 2.

**Figure 3:**
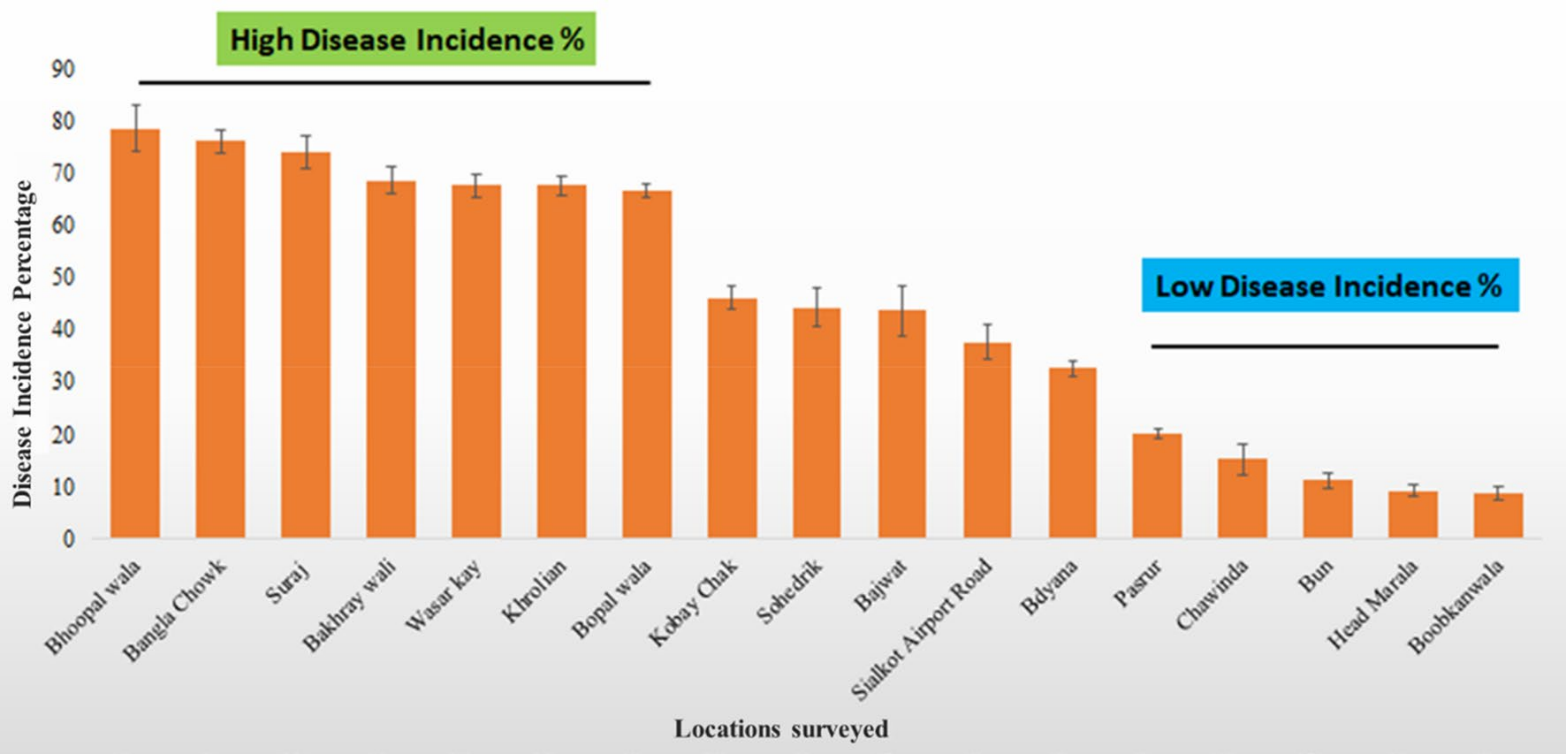
The highest and lowest maize stalk rot disease incidence and severity were recorded from the surveyed locations.

Symptoms of BSRM were visually recorded from the visited fields. Data on *D*_*I*_ and *D*_*S*_ showed variations in disease occurrence levels within the villages of all districts in the Sialkot region. Out of 226 visited locations, the highest *D*_*I*_ and *D*_*S*_ were recorded from Bhoopal Wala (78.5 ± 4.5), Bangla Chowk (76 ± 2.3), Suraj (74 ± 3.2), Bhakhrewali (68.5 ± 2.7), Warsalke (67.5 ± 2.3), Khrolian (67.5 ± 1.8). Whereas, low *D*_*I*_ and *D*_*S*_ were recorded in Pasrur (20 ± 1), Chawinda (15 ± 2.9), Bun (11 ± 1.4), Head Marala (9 ± 1.1), and Boobkanwala (8.5 ± 1.4) (Table S2). Areas showing the highest and lowest *D*_*I*_ percentages are presented in Fig. 3.

### Biochemical characterization of *Dickeya zeae*

Different tests such as catalase test, motility test, indole formation, H_2_S production, gelatin liquefaction, nitrate reduction, starch hydrolase, urease activity, and levan production were performed for the biochemical characterization of *D. zeae* (Table 2). All bacterial isolates (ERCAR-1, ERCAR-2, ERCAR-3, ERCAR-4, ERCAR-5) were found positive for motility test, able to perform catalase activity and nitrate reduction, but all strains resulted negative for H_2_S production and urease activity. For indole formation test results were negative with isolates ERCAR-1, ERCAR-2, ERCAR-5, but this assay was not done for ERCAR-3 and ERCAR-4. Of all the tested bacteria displayed gelatin liquefaction ability, except ERCAR-5 which was not tested. Starch hydrolase test results were negative for ERCAR-1, ERCAR-3, ERCAR-4, ERCAR-5 while the test was not performed for ERCAR-2. Similarly, bacterial isolates such as ERCAR-1, ERCAR-2, ERCAR-3, ERCAR-5 were observed negative for levan production while the test was not performed for ERCAR-4.

**Table 2:** Biochemical characterization of *Dickeya zeae* strains from Sialkot District, Pakistan, associated with stalk rot of maize.

Biochemical Assay	ERCAR-1	ERCAR-2	ERCAR-3	ERCAR-4	ERCAR-5
motility test	+	+	+	+	+
Indole formation	−	−	ND	ND	−
H_2_S production	−	−	−	−	−
Gelatin liquefaction	+	+	+	+	ND
Catalase activity	+	+	+	+	+
Nitrate reduction	+	+	+	+	+
Starch hydrolase	−	ND	−	−	−
Urease activity	−	−	−	−	−
Levan production	−	−	−	ND	−

+ = Positive test results; − = Negative test results; ND = Not done.

### Environmental variable selection and model performance

**Figure 4:**
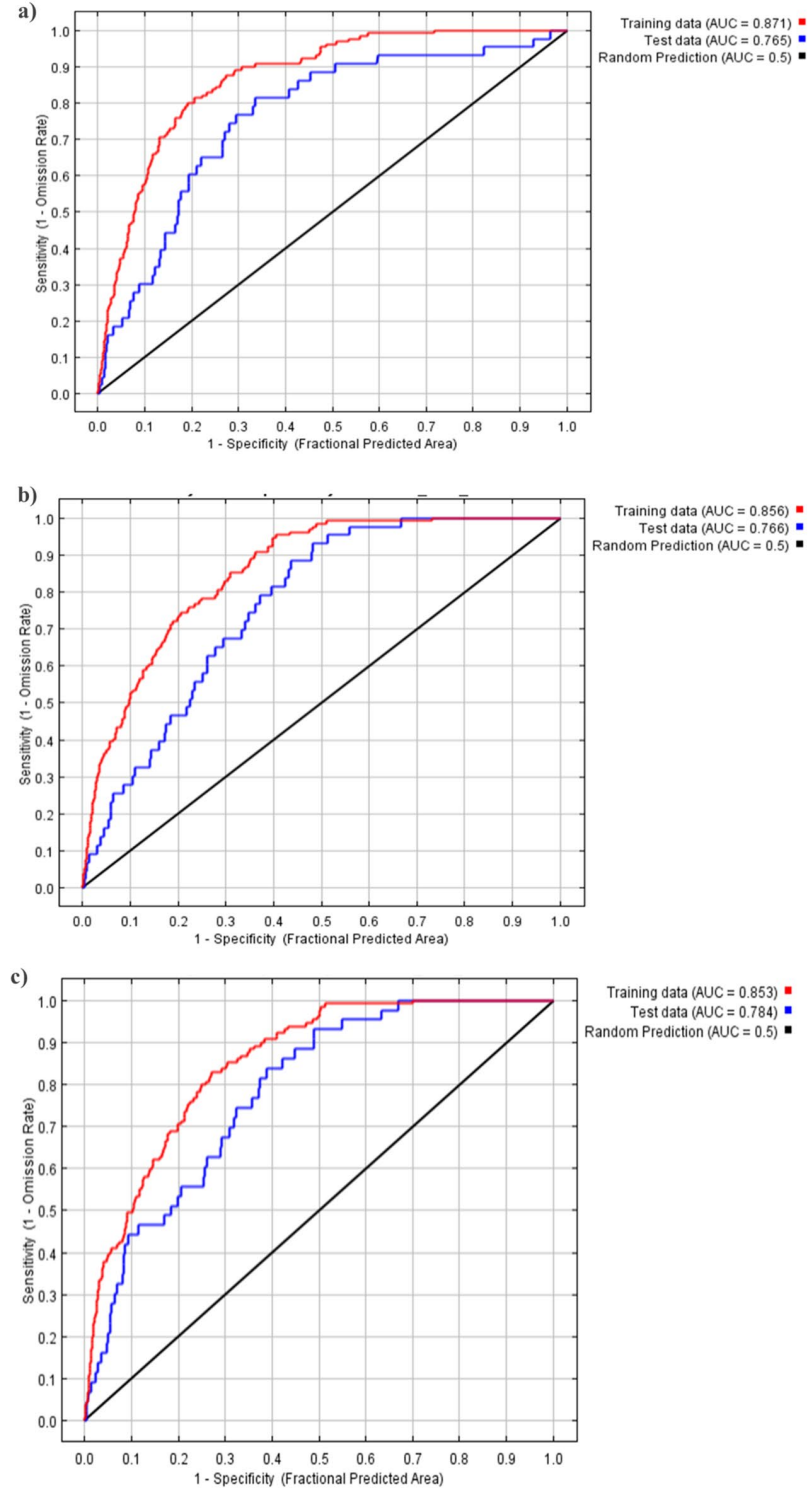
Change in receiver operating characteristic curve for (**a**) current year, (**b**) 2050, and (**c**) 2070.

Pearson correlation revealed that coefficients between 28 sets of the 19 variables were higher than 0.8. Nineteen variables were selected based on the initial model percentage contribution. Pearson correlation value between every two variables was < 0.8, and the rate of the total contribution of all other variables was 100%. Currently, the most contributing variable bio-16 with a contribution of 25%, followed by bio-2 (11.5%), bio-4 (9.5%), and bio-1 (8.6%) while minimal contributing factors were bio-14 (0.3%), followed by bio-12 (0.1%) and bio-7 with a contribution of 0%. An omission curve on the training data showed a trend similar to the test data. The AUC of the training and test data were 0.871 and 0.765, respectively in the current scenario as shown in Fig. 4a. The accuracy of the current distribution model was observed excellent based on the model evaluation index.

For 2050, the most contributing variable was observed in annual precipitation (bio-12) with a contribution of 29.1%, followed by bio-8 (20.6%), bio-14 (11.1%), and bio-3 (8.3%) while minimal contributing factors were bio-15 (0.3%), followed by bio-7 (0.2%) and the least contributing factor was bio-10 with a contribution of 0.1%. The AUC of the training and test values were 0.856 and 0.766, respectively in 2050 as shown in Fig. 4b.

In the case of 2070, the most contributing variable was bio-12 with a contribution of 29.9%, followed by bio-8 (22.4%), bio-14 (13.6%), and bio-17 (8.6%) while minimal contributing factors were bio-2 (0.3%), and the least contributing factors were bio-15 and bio-16 with 0% contribution. The AUC of the training and test values were 0.853 and 0.784, respectively in the 2070 distribution model as shown in Fig. 4c.

### Relationship between the occurrence of *Dickeya zeae* and bioclimatic variables

#### Current year

**Figure 5:**
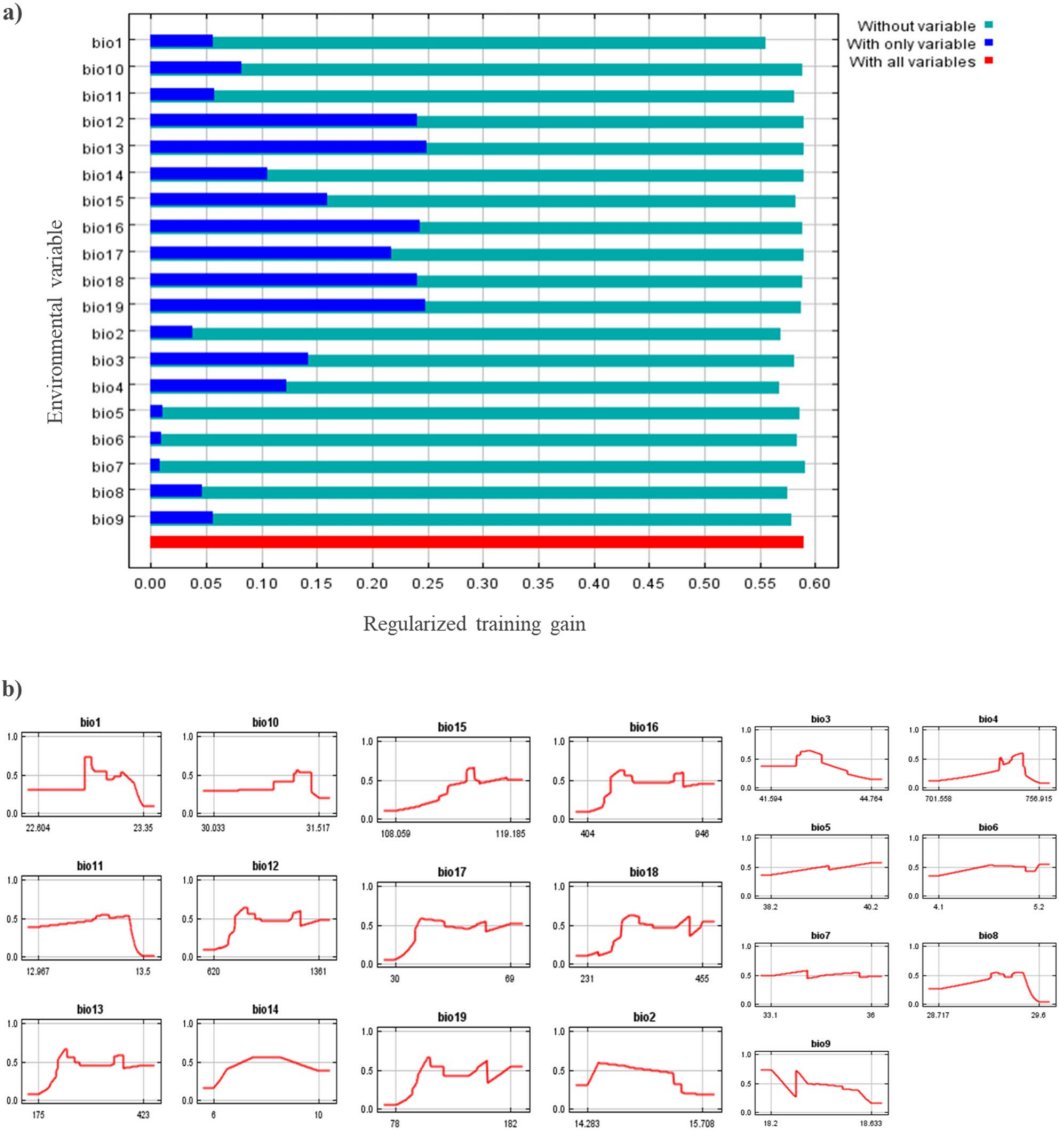
(**a**) Jackknife test of bioclimatic variables which are influential in the distribution of *D. zeae* in the current year; (**b**) Response curves of the bioclimatic variables showing how each variable affects the Maxent Prediction for the current year.

Jackknife analysis displayed that the environmental variable with the highest gain when used in isolation is bio-13 among all bioclimatic layers. Similarly, annual precipitation (bio-12), precipitation seasonality (bio-15), precipitation of wettest quarter (bio-17), and precipitation of warmest quarter (bio-18), were the most influential environmental variables that affect the distribution of *D. zeae,* with a training gain as presented in Fig. 5a. Precipitation of the coldest quarter (bio-19) and mean temperature of the warmest quarter (bio-10), were also influential bioclimatic variables with training gain. The response curve thresholds of the variables are given in Fig. 5b. Bio-16 ranged from 404 to 946 mm, bio-2 ranged from 14.2 to 15.7 °C, bio-4 ranged from 701 to 756 °C, bio-1 ranged from 22.6 to 23.3 °C, bio-18 ranged from 231 to 455 mm and bio-3 ranged from 41.5 to 44.7 °C while bio-19 ranged from 78 to 182 mm, bio-10 ranged from 30.0 to 31.5 °C, bio-5 ranged from 38.2 to 40.2 °C, bio-6 ranged from 4.1 to 5.2 °C, bio-7 ranged from 31.1 to 36 °C, bio-8 ranged from 28.7 to 29.6 °C, bio-9 ranged from 18.2 to 18.6 °C, bio-10 ranged from 30.0 to 31.5 °C, bio-11 ranged from 12.9 to 13.5 °C, bio-12 ranged from 620 to 1361 mm, bio-13 ranged from 175 to 423 mm, bio-14 ranged from 6 to 10 mm, bio-15 ranged from 108.0 to 119.1 mm, bio-17 ranged from 30 to 69 mm and bio-19 ranged from 78 to 182 mm.

#### For 2050

**Figure 6:**
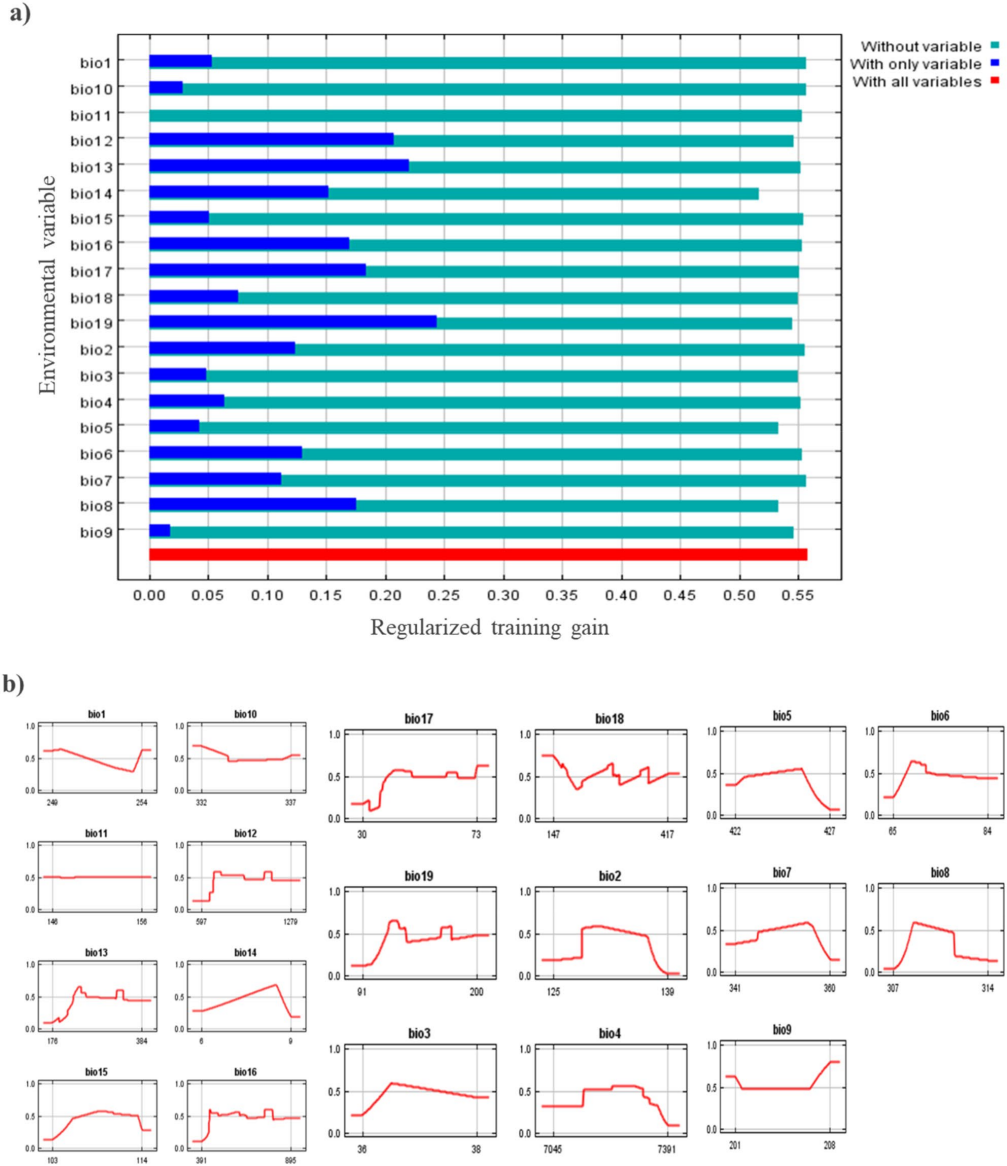
(**a**) Jackknife test of bioclimatic variables which are influential in the distribution of *D. zeae* for 2050; (**b**) Response curves of the bioclimatic variables showing how each variable affects the Maxent Prediction for 2050.

Jackknife analysis showed that the environmental variable with the highest gain when used in isolation is bio-19 among all bioclimatic layers, which appears to reflect the most useful information by itself. Jackknife's analysis revealed that the omission of bio-14 decreases the gain the most. Which seems to have maximum information that is not present in the other variables.Similarly, mean temperature of wettest quarter (bio-8) annual precipitation (bio-12), precipitation of wettest month (bio-13), precipitation of the driest month (bio-14), precipitation of seasonality (bio-15), precipitation of wettest quarter (bio-16), and precipitation of the driest quarter (bio-17), were the most influential variables that affect the distribution of *D. zeae,* with a training gain as presented in Fig. 6a. The response curve thresholds of the variables are given in Fig. 6b. The bioclimatic variable values were as follows: bio-16 ranged from 391 to 895 mm, bio-2 ranged from 125 to 139 °C, bio-4 ranged from 7045 to 7391 °C, bio-1 ranged from 249 to 254 °C, bio-18 ranged from 147 to 417 mm and bio-3 ranged from 36 to 38 °C while bio-19 ranged from 91 to 200 mm, bio-10 ranged from 332 to 337 °C, bio-5 ranged from 422 to 427 °C, bio-6 ranged from 65 to 84 °C, bio-7 ranged from 341 to 380 °C, bio-8 ranged from 307 to 314 °C, bio-9 ranged from 201 to 208 °C, bio-10 ranged from 332 to 337 °C, bio-11 ranged from 146 to 156 ^◦^C, bio-12 ranged from 597 to 1279 mm, bio-13 ranged from 176 to 384 mm, bio-14 ranged from 6 to 9 mm, bio-15 ranged from 103 to 114 mm, bio-17 ranged from 30 to 73 mm and bio-19 ranged from 91 to 200 mm.

#### For 2070

**Figure 7:**
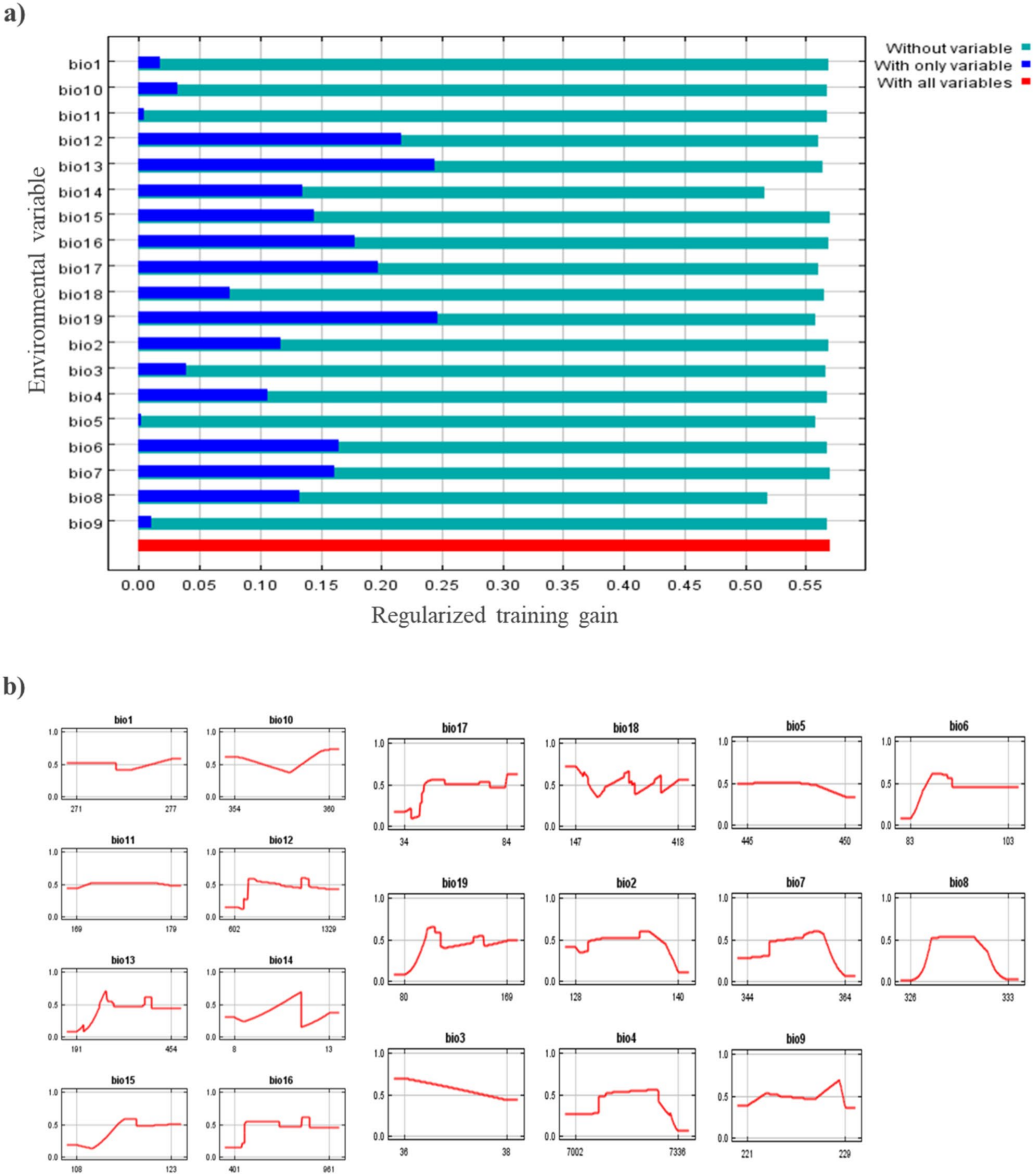
(**a**) Jackknife test of bioclimatic variables which are influential in the distribution of *D. zeae* in 2070; (**b**) Response curves of the bioclimatic variables showing how each variable affects the Maxent Prediction for 2070.

In the case of 2070, Jackknife's analysis revealed that the omission of bio-14 decreases the gain the most. Which seems to have maximum information that is not present in the other variables. However, annual precipitation (bio-12), precipitation of wettest month (bio-13), precipitation of wettest quarter (bio-16), precipitation of driest quarter (bio-17), and precipitation of the coldest quarter (bio-19), were the most influential factors affecting the distribution of *D. zeae*, with a training gain as presented in Fig. 7a. Response curve thresholds of the variables are presented in Fig. 7b. The bioclimatic variable values were as follows: bio-16 ranged from 401 to 961 mm, bio-2 ranged from 128 to 140 °C, bio-4 ranged from 7002 to 7336 °C, bio-1 ranged from 271 to 277 °C, bio-18 ranged from 147 to 418 mm and bio-3 ranged from 36 to 38 °C while bio-19 ranged from 80 to 169 mm, bio-10 ranged from 354 to 360 °C, bio-5 ranged from 445 to 450 °C, bio-6 ranged from 83 to 103 °C, bio-7 ranged from 344 to 364 °C, bio-8 ranged from 326 to 333 °C, bio-9 ranged from 221 to 229 °C, bio-10 ranged from 354 to 360 °C, bio-11 ranged from 169 to 179 °C, bio-12 ranged from 602 to 1329 mm, bio-13 ranged from 191 to 454 mm, bio-14 ranged from 8 to 13 mm, bio-15 ranged from 108 to 123 mm, bio-17 ranged from 34 to 84 mm and bio-19 ranged from 80 to 169 mm.

#### Modeled distribution and conservation assessment of *Dickeya zeae*

**Figure 8:**
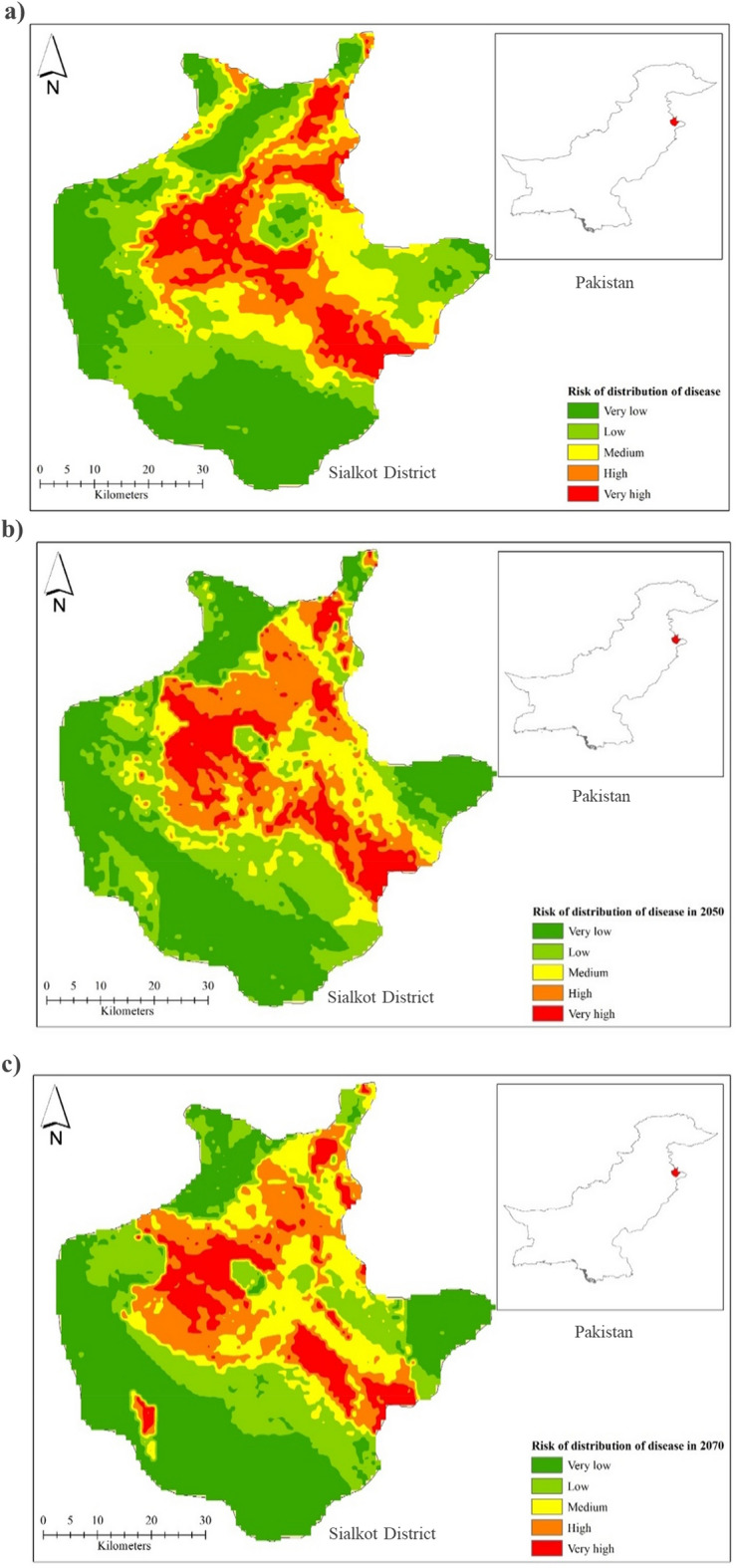
Risk of bacterial stalk rot of maize disease distribution: (**a**) current, (**b**) 2050, and (**c**) 2070.

High suitability areas out of 226 visited locations in the Sialkot region were Bhoopal Wala, Bangla Chowk, Suraj, Bhakhrewali, Warsalke, Khrolian, and Bopal Wala showed the highest *D*_*I*_ and *D*_*S*_. Moderate suitability was located in Kobay Chak, Sohedrik, Bajwat, Sialkot Airport Road, and Bdyana. While low-suitability areas were located in Pasrur, Chawinda, Bun, Head Marala, and Boobkanwala. The total suitability area trend increased over time from the current to the 2050s and 2070s. The current distribution of *D. zeae* increased in the low suitability area, as well as the moderate suitability areas while the trend in the 2050s and 2070s will also increase as shown in Fig. 8.

**Figure 9:**
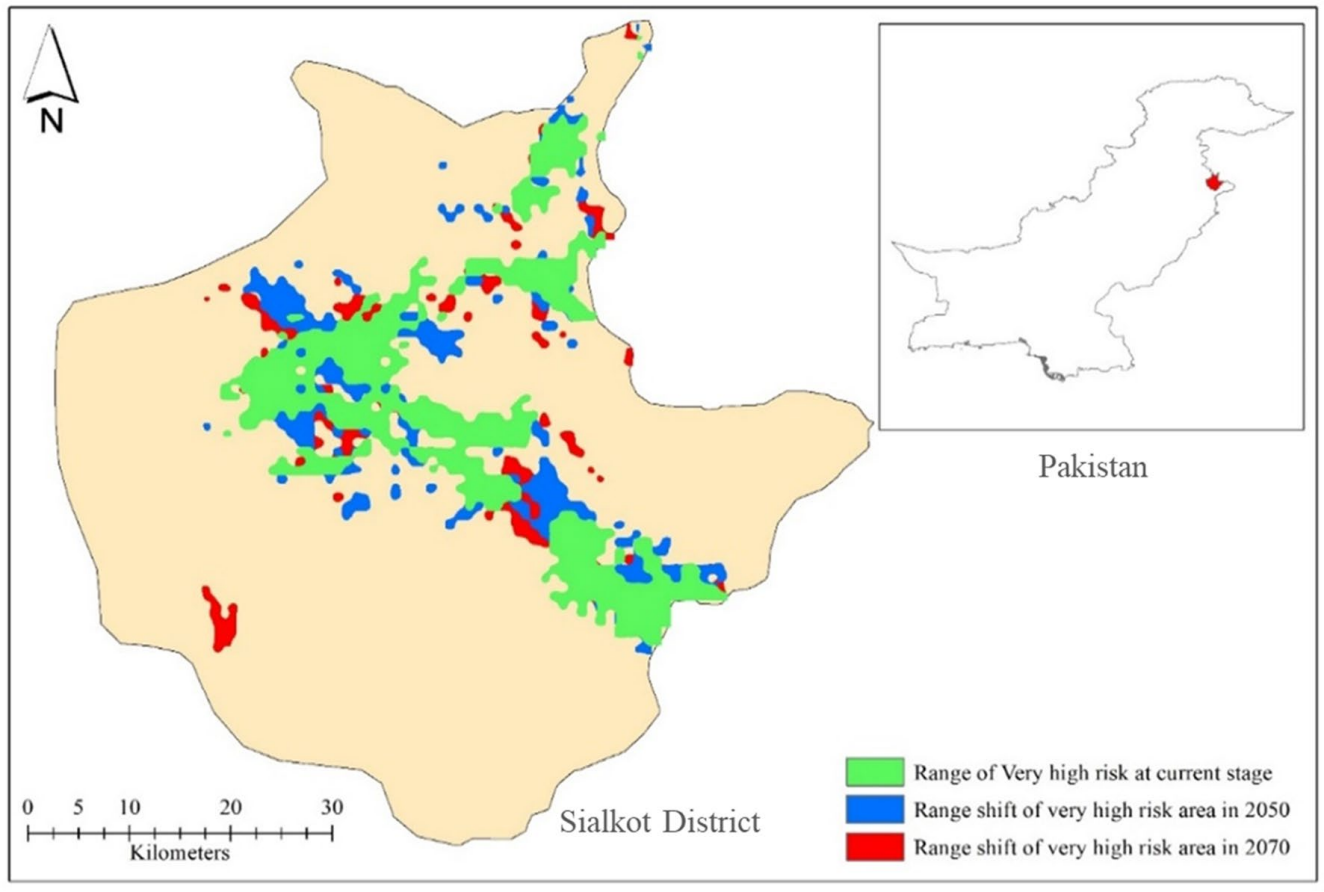
Overlapping map of the ranges of very high risk of BSRM in the current scenario, 2050 and 2070 predictions.

The overlapping suitability regions were also studied by overlapping the present and future (2050s and 2070s) potential distributions of *D. zeae* as shown in Fig. 9. The overlapping map indicated that there are chances that the area of BSRM disease progression will increase over the years. The green color indicates a very high risk at the current stage, the blue color indicates a very high risk in the 2050s while the red color indicates a very high risk in the 2070s. Bhoopal wala, Bangla Chowk, Suraj, Bhakhrewali, Warsalke, Khrolian, and Bopal wala were the high suitability areas, with high *D*_*I*_ and *D*_*S*_ 70–80%. Kobay Chak, Sohedrik, Bajwat, Sialkot Airport Road, and Bdyana were the moderate suitability areas, with 30–50% *D*_*I*_ and *D*_*S*_. Pasrur, Chawinda, Bun, Head Marala, and Boobkanwala were the low suitability areas, with 10–20% *D*_*I*_ and *D*_*S*_.

## Discussions

Maize production is vulnerable to the attack of various pathogens resulting into severe yield losses. Of all the major diseases, BSRM caused by *D. zeae* is of great concern because of significant yield losses. *D. zeae* is the most destructive plant pathogen that infects maize plants at any growth stage. A comprehensive field survey reflected that out of 226 visited sites in the district Sialkot region, DI ranged from 8.5 to 76% and this variation in the level of disease occurrence could be due to the cultivation of a wide variety of maize hybrids, environmental conditions prevailing in the region, crops rotation and disease management and cultural practices^56^. Similarly, in another related study, Sharma et al.^57^ recorded that the grain yield losses due to BSRM ranged from 21 to 98%^57^. In a study, Kumar et al.^13^ narrated that under favorable conditions, *D. zeae* could result in 98.8% crop yield losses, and the wide prevalence and survival of the bacterium is due to the wide host range, high temperature, and moisture (rainy season)^13^. Our results are similar to the study of Tahir et al.^30^ where eight maize-growing districts in central and south Punjab, Pakistan were surveyed for BSRM^30^. Results demonstrated that all the surveyed areas showed 100% prevalence and the highest *D*_*I*_ and *D*_*S*_ up to 53% and 30.2% respectively.

In the present study, the biochemical attributes of *D. zeae*, our results contrast with the findings of Jatoth et al.^56^ where all the tested bacterial strains displayed positive responses for H_2_S production and indole formation^56^, meanwhile the strains from the present study, resulted negative in the production of these compounds. Similarly, Kaur et al.^58^ subjected bacterial isolates to various biochemical tests and found them to show different reactions for utilization of starch and other sugars, gelatin liquefication, and growth at high salt levels^58^. Biochemical characterization helps to identify bacterial species based on the differential biochemical activities; nevertheless, various strains of *D. zeae* might demonstrate differences in various biochemical characteristics because of alterations in specific enzymatic activities^58^.

The Maxent algorithm modeling was employed to predict areas at high risk of disease outbreaks based on occurrence and environmental data in Sialkot; the model was further used to forecast the disease outbreaks in the coming years. In our study, results showed that a total of nineteen bioclimatic variables were selected that were slightly different from the variables used in other studies^59–61^.

Although, the distribution and presence of pathogens may depend upon many factors, here we only focused on bioclimatic factors, because, these factors are biologically meaningful in defining the species distribution^62^. In many previous studies, these factors were selected for studying the habitats and their implications for the conservation of species as well as for the development of policies^20,21, 44^. Bioclimatic variables used for modeling by Maxent software have already reported^45^. Certain variables play a significant role in the distribution of stalk rot of maize. Currently, the precipitation of the wettest quarter, mean diurnal range, temperature seasonality, and annual mean temperature made significant contributions to the model. The AUC of the training and test data were 0.871 and 0.765, respectively in the current distribution model. Jackknife analysis revealed that the environmental variable with the highest gain when used in isolation was bio-13 among all bioclimatic layers.

In the coming years in 2050 the variables of annual precipitation, mean temperature of the wettest quarter, precipitation of the driest month, and isothermality will be crucial. In the 2070s, it was predicted that certain variables such as annual precipitation, mean temperature of wettest quarter, precipitation of driest month, and precipitation of driest quarter would be influential in a disease outbreak. A similar study explained the connection between bioclimatic variables and the potential distribution of *Haemaphysalis spinigera* associated with Kyasanur forest disease. The study concluded that the average temperature of the warmest quarter, average diurnal temperature range, precipitation of the wettest period, and annual precipitation contribute to affecting the spatial distribution of *H. spinigera*^63^.

High suitability areas were Bhoopal wala, Bangla Chowk, Suraj, Bhakhrewali, Warsalke, Khrolian, and Bopal wala showed the highest *D*_*I*_ and *D*_*S*_. Moderate suitability was in Kobay Chak, Sohedrik, Bajwat, Sialkot Airport Road, and Bdyana. While low suitability areas were in Pasrur, Chawinda, Bun, Head Marala, and Boobkanwala. The total suitability area trend increased over time from the current to the 2050s and 2070s. The current distribution of *D. zeae* increased in the low suitability area, as well as the moderate suitability areas while the trend in 2050s and 2070s will also increase. The suitability regions were also identified by overlapping current and future (2050s and 2070s) potential distributions of *D. zeae*. The overlapping map indicated that there are chances that the area of BSRM disease progression will increase over the years. In another study, environmental conditions linked with the *Fusarium solani* optimum inoculum density for disease occurrence were accessed and a strong convergence on the environmental requirements of both the host and the disease progress was observed. Among different variables, precipitation and temperature variables were found important for explaining the disease spread^64^. These results were also similar to the findings of Sallam et al.^65^ where ecological niche modeling and land cover risk regions for rift valley fever vector, *Culex tritaeniorhynchus* Giles in Jazan, Saudi Arabia were identified^65^. Another study in Saudi Arabia on ecological distribution modeling of two malaria mosquito vectors by GIS showed similar results^19^. The same parameters were used for working with climate data and niche modeling^66^.

Here, we constructed the maps on the potential distribution of BSRM in Sialkot, Pakistan and these maps can effectively be used in furcating and disease management strategies development. Moreover, our work may be used as a reference to predict other phytopathogenic diseases in major crops and ensure food security.

## Conclusion

Bacterial stalk rot of maize caused by *D. zeae* causes significant yield losses. In this work, isolates of *D. zeae* from the Sialkot region in Pakistan, were characterized biochemically. During extensive fields surveys, disease incidence was recorded, ranging from 43.5 to 78.5%. Maxent algorithm modeling revealed the areas at risk of disease outbreaks, which also predicted a high risk of BSR outbreak in 2050 and 2070. These results are very important for predicting the spatial distribution trends of BSRM in accordance with the climatic conditions in the coming years. Our findings will be useful in developing and adopting integrated disease management programs to prevent the spreading and control of BSR disease in maize crops.

### Supplementary Information

Supplementary Tables.

## Data Availability

The datasets analysed during this study are included in this manuscript.
