# Supplementary material for: Impact of climate change on potential distribution of Dickeya zeae causal agent of stalk rot of maize in Sialkot district Pakistan
Source: Sci Rep. 2024 Jan 31;14:2614. doi: 10.1038/s41598-024-52668-2 (PMC10830500; doi:10.1038/s41598-024-52668-2)
Supplement: Supplementary file 1 — Supplementary Tables. [file 41598_2024_52668_MOESM1_ESM.docx]

**Supplementary Material.**

**Table S1.** List of visited maize growing locations and their GPS coordinates.

| **Sr. No** | **Area Name** | **GPS Coordinate** |
| --- | --- | --- |
|  | Shahzada | N32º17’57.8” E074º44’32.1” |
|  | Shahzada | N32º17’57.5” E074º44’10.1” |
|  | Shahzada | N32º17’46.1” E074º44’55.2” |
|  | Shahzada | N32º17’26.8” E074º44’51.3” |
|  | Shahzada | N32º18’03.3” E074º44’47.4” |
|  | Shahzada | N32º18’04.1” E074º45’05.0” |
|  | Kotli | N32º18’11.7” E074º44’03.7” |
|  | Kapoor pur | N32º18’14.7” E074º43’21.8” |
|  | Kamal pur | N32º18’40.5” E074º43’29.9” |
|  | Kamal pur | N32º18’58.4” E074º43’28.3” |
|  | Nawa pind | N32º19’05.5” E074º43’00.6” |
|  | Chota Shahzada | N32º17’30.8” E074º44’27.2” |
|  | Waain | N32º17’19.5” E074º43’45.5” |
|  | Chak Raja | N32º17’15.6” E074º43’20.6” |
|  | Dugri | N32º16’37.7” E074º43’10.3” |
|  | Essa | N32º16’54.5” E074º42’41.9” |
|  | Chaiye wali | N32º16’47.1” E074º42’05.8” |
|  | Chuhan | N32º16’26.0” E074º41’10.0” |
|  | Pasrur | N32º16’14.5” E074º40’38.1” |
|  | Pasrur | N32º16’54.8” E074º39’11.0” |
|  | Dhoal | N32º18’12.4” E074º45’20.3” |
|  | Kotli sayeda | N32º19’07.6” E074º45’21.8” |
|  | Rajiyan | N32º19’29.8” E074º44’28.8” |
|  | Nawa pind | N32º19’58.3” E074º44’10.3” |
|  | Chawinda | N32º20’45.7” E074º43’45.8” |
|  | Chawinda | N32º21’00.9” E074º41’34.8” |
|  | Jasuran | N32º21’06.7” E074º41’08.5” |
|  | Jasuran | N32º21’17.9” E074º40’15.5” |
|  | Jasuran | N32º21’22.1” E074º39’54.2” |
|  | Sohedriky | N32º21’33.9” E074º38’55.4” |
|  | Sohedriky | N32º21’32.7” E074º38’29.8” |
|  | Badhyana | N32º21’56.8” E074º37’40.4” |
|  | Badhyana | N32º22’41.6” E074º36’47.0” |
|  | Badhyana | N32º22’40.4” E074º36’15.8” |
|  | Badhyana | N32º23’44.1” E074º36’16.1” |
|  | Badhyana | N32º23’14.5” E074º36’37.6” |
|  | Saho chuk | N32º22’41.2” E074º35’26.5” |
|  | Badi pur | N32º22’40.3” E074º34’39.4” |
|  | Nano wali | N32º22’57.0” E074º34’29.6” |
|  | Guri malihan | N32º23’30.9” E074º34’37.1” |
|  | Warion | N32º24’17.0” E074º34’46.2” |
|  | Warion | N32º24’41.8” E074º35’15.7” |
|  | Bhutar | N32º21’54.1” E074º37’13.8” |
|  | Purab | N32º21’15.6” E074º37’38.5” |
|  | Bhati | N32º20’46.4” E074º37’33.7” |
|  | Nakhy | N32º20’01.1” E074º38’01.7” |
|  | Langy | N32º19’13.0” E074º38’27.7” |
|  | Manjhky | N32º18’41.5” E074º38’48.6” |
|  | Mali pur | N32º17’37.9” E074º39’01.3” |
|  | Nokrian | N32º16’30.8” E074º41’04.4” |
|  | Handle pura | N32º31’36.2” E074º31’37.2” |
|  | Kubay chak | N32º35’16.9” E074º33’44.9” |
|  | Kubay chak | N32º35’49.9” E074º33’47.8” |
|  | Pindi panjora | N32º36’37.2” E074º34’00.7” |
|  | Pindi panjora | N32º37’37.7” E074º34’32.2” |
|  | Rum Adda | N32º38’14.7” E074º34’53.3” |
|  | Rum Adda | N32º38’37.2” E074º34’54.3” |
|  | Seedra khrud | N32º39’20.5” E074º34’59.5” |
|  | Khojay chak | N32º42’12.8” E074º36’02.4” |
|  | Bajwat | N32º40’58.0” E074º35’07.2” |
|  | Bajwat | N32º41’24.4” E074º35’12.7” |
|  | Bajwat | N32º42’48.7” E074º36’12.0” |
|  | Bajwat | N32º43’07.0” E074º36’43.9” |
|  | Bajwat | N32º42’51.3” E074º36’18.1” |
|  | Bajwat | N32º43’21.8” E074º37’05.5” |
|  | Bajwat | N32º44’14.7” E074º37’43.9” |
|  | Bajwat | N32º43’49.1” E074º36’57.5” |
|  | Bajwat | N32º44’07.2” E074º37’35.6” |
|  | Bajwat | N32º42’08.2” E074º35’34.3” |
|  | Bajwat | N32º41’29.8” E074º35’16.5” |
|  | Bajwat | N32º40’58.2” E074º35’07.4” |
|  | Ugoke | N32º29’09.0” E074º27’56.4” |
|  | Ugoke | N32º28’45.3” E074º25’58.2” |
|  | Ugoke | N32º28’39.2” E074º24’28.1” |
|  | Wazirabad road | N32º28’42.1” E074º23’31.9” |
|  | Sialkot Airport Road | N32º29’34.2” E074º22’29.7” |
|  | Sialkot Airport Road | N32º30’06.4” E074º22’32.0” |
|  | Sialkot Airport Road | N32º30’34.5” E074º22’29.1” |
|  | Sialkot Airport Road | N32º31’35.2” E074º22’16.8” |
|  | Chak jeetea | N32º32’10.5” E074º23’04.5” |
|  | Abiala | N32º32’31.5” E074º23’40.9” |
|  | Mareeq pura | N32º29’09.6” E074º20’43.5” |
|  | Mareeq pura | N32º29’22.3” E074º20’41.8” |
|  | Karnanwali | N32º30’13.2” E074º20’53.6” |
|  | Karnanwali | N32º30’46.6” E074º21’02.2” |
|  | Rao ghun | N32º30’54.0” E074º21’32.7” |
|  | Jugat pur | N32º31’10.7” E074º21’46.3” |
|  | Firdoos pura | N32º27’54.1” E074º20’49.8” |
|  | Kot dhara daa | N32º26’58.5” E074º20’09.9” |
|  | Nunaan wali kotli | N32º26’34.4” E074º21’19.6 |
|  | Bhoopal wala | N32º25’34.4” E074º21’42.6” |
|  | Bhoopal wala | N32º25’12.6” E074º21’46.0” |
|  | Wasarki adda | N32º24’32.3” E074º21’51.2” |
|  | Warsalke | N32º24’10.8” E074º21’45.1” |
|  | Adamkay Cheema | N32º23’40.5” E074º21’39.8” |
|  | Adamkay Cheema | N32º23’18.0” E074º21’35.5” |
|  | Wasan kay | N32º22’52.0” E074º21’31.0” |
|  | Daska | N32º21’46.0” E074º21’25.4” |
|  | Daska | N32º20’39.0” E074º23’09.6” |
|  | Daska | N32º21’19.5” E074º23’28.6” |
|  | Sambrial Airport Road | N32º30’47.4” E074º22’25.1” |
|  | Deeto wali | N32º33’59.1” E074º22’08.8” |
|  | Channi gondal | N32º34’04.9” E074º21’17.8” |
|  | Deen pura | N32º34’04.0” E074º20’45.6” |
|  | Bhakhrewali | N32º34’42.3” E074º18’13.9” |
|  | Motra Bdyana road | N32º23’14.7” E074º36’38.0” |
|  | Warion | N32º24’16.6” E074º34’49.1” |
|  | Dulam Ghazzi | N32º25’24.6” E074º33’08.9” |
|  | Dulam Ghazzi | N32º27’02.0” E074º33’28.9” |
|  | Bhagowaal | N32º26’00.3” E074º39’00.3” |
|  | Bakarpur | N32º33’52.0” E074º29’45.6” |
|  | Randhawa | N32º17’57.9” E074º34’47.8” |
|  | Kotli kurlan | N32º17’46.7” E074º29’47.2” |
|  | Lalar | N32º17’56.1” E074º28’56.7” |
|  | Kotli nonan | N32º26’23.5” E074º21’22.0” |
|  | Miani | N32º39’47.5” E074º30’48.3” |
|  | Dilawarpur | N32º39’51.9” E074º34’54.4” |
|  | Khojay chak | N32º40’51.7” E074º35’06.5” |
|  | Najwal | N32º43’21.7” E074º37’05.4” |
|  | Chakpondra | N32º49’17.3” E074º41’20.2” |
|  | Sadra | N32º38’45.2” E074º34’52.8” |
|  | Chand Bagh | N32º31’39.5” E074º28’32.2” |
|  | Kapoor wali | N32º31’52.9” E074º27’32.2” |
|  | Kapoor wali | N32º32’11.0” E074º27’32.7” |
|  | Kapoor wali | N32º32’34.4” E074º27’32.0” |
|  | Kapoor wali | N32º32’15.8” E074º26’27.2” |
|  | Kala kumra | N32º32’51.0” E074º27’42.0” |
|  | Sandhu wala | N32º32’59.2” E074º27’59.4” |
|  | Sandhu wala | N32º33’19.3” E074º28’28.2” |
|  | Laal pur | N32º33’24.2” E074º29’21.8” |
|  | Shah Block | N32º32’34.8” E074º25’44.6” |
|  | Khumbranwala | N32º32’51.9” E074º25’13.5” |
|  | Khumbranwala | N32º32’57.4” E074º24’31.1” |
|  | Khumbranwala | N32º33’07.3” E074º24’32.1” |
|  | Abiyala | N32º32’37.2” E074º23’54.8” |
|  | Tanbray Basir | N32º33’52.4” E074º23’13.9” |
|  | Suraj | N32º33’42.9” E074º22’56.1” |
|  | Bangla Chowk | N32º33’59.7” E074º22’07.2” |
|  | Urah Chowk | N32º29’13.3” E074º35’29.3” |
|  | Syedan wali | N32º29’04.0” E074º35’42.3” |
|  | Syedan wali | N32º28’23.6” E074º35’27.6” |
|  | Raey pur | N32º28’11.3” E074º35’07.3” |
|  | Gopal pur | N32º27’54.0” E074º35’50.8” |
|  | Pathan wali | N32º27’10.6” E074º36’56.4” |
|  | Ledhoki | N32º26’45.0” E074º37’42.0” |
|  | Ledhoki | N32º26’27.9” E074º38’00.1” |
|  | Rang pur | N32º35’55.4” E074º38’00.1” |
|  | Dhuli | N32º34’37.1” E074º38’07.6” |
|  | Malany | N32º34’50.9” E074º37’24.1” |
|  | Plura | N32º35’01.1” E074º36’08.2” |
|  | Romal Jattan | N32º35’53.2” E074º36’17.6” |
|  | Najwal | N32º36’31.4” E074º36’34.7” |
|  | Kobay Chak | N32º35’42.7” E074º33’31.1” |
|  | Kammanwala | N32º32’39.1” E074º36’02.5” |
|  | Malhappar | N32º32’50.4” E074º38’04.4” |
|  | Kajlial | N32º33’42.2” E074º40’22.6” |
|  | Malagarpur | N32º37’20.5” E074º36’44.4” |
|  | Jhun | N32º33’25.1” E074º38’03.7” |
|  | Change | N32º34’02.5” E074º37’46.4” |
|  | Doburji Mallian | N32º28’13.5” E074º31’20.8” |
|  | Butter | N32º28’02.4” E074º30’28.7” |
|  | Pakki Kotli | N32º27’20.9” E074º30’36.8” |
|  | Miani | N32º27’19.2” E074º30’01.3” |
|  | Sadra Badra | N32º27’14.4” E074º30’04.3” |
|  | Addha | N32º26’37.9” E074º29’05.2” |
|  | Beera | N32º26’18.1” E074º29’22.8” |
|  | Hapu garaha | N32º26’25.8” E074º30’06.8” |
|  | Ghuinke | N32º25’29.2” E074º27’26.4” |
|  | Kotli Bagho | N32º25’32.2” E074º26’52.1” |
|  | Dhand pur | N32º25’32.6” E074º26’24.2” |
|  | Khrolian | N32º25’31.8” E074º26’06.5” |
|  | Raliyo kay | N32º25’29.5” E074º25’05.9” |
|  | Raliyo kay | N32º25’27.0” E074º24’42.8” |
|  | Sahib kay | N32º25’34.0” E074º24’16.7” |
|  | Badho kay Cheema | N32º25’27.3” E074º23’58.8” |
|  | Ghuinke | N32º25’37.7” E074º27’22.6” |
|  | Mandyianwala | N32º24’49.2” E074º34’17.0” |
|  | Walany | N32º24’44.7” E074º33’47.4” |
|  | Satowali | N32º25’22.9” E074º33’08.6” |
|  | Satowali | N32º25’50.8” E074º33’16.7” |
|  | Chouni Slehrian | N32º26’50.5” E074º33’30.8” |
|  | Pico shore | N32º27’25.8” E074º33’22.2” |
|  | Kalan Joudhay wali | N32º27’51.7” E074º34’24.8” |
|  | Ghalotian Mor | N32º17’58.5” E074º18’43.6” |
|  | Ghalotian Road | N32º16’58.7” E074º18’57.0” |
|  | Ghalotian Road | N32º16’35.1” E074º19’05.5” |
|  | Ghalotian Road | N32º16’53.6” E074º18’44.1” |
|  | Ghalotian Road | N32º16’57.1” E074º18’25.3” |
|  | Ghalotian Road | N32º16’54.5” E074º18’20.6” |
|  | Musalmanian | N32º16’22.5” E074º17’27.2” |
|  | Musalmanian | N32º16’23.1” E074º17’26.3” |
|  | Musalmanian | N32º16’24.9” E074º17’26.5” |
|  | Musalmanian | N32º16’20.6” E074º17’23.4” |
|  | Musalmanian | N32º16’25.1” E074º17’22.7” |
|  | Othian | N32º15’55.9” E074º16’42.6” |
|  | Boobkan wala | N32º17’41.1” E074º18’26.8” |
|  | Gujranwala Road | N32º17’58.4” E074º18’39.1” |
|  | Gujranwala Road | N32º18’04.0” E074º18’45.8” |
|  | Mandrawala | N32º21’27.9” E074º21’24.6” |
|  | Mandrawala | N32º21’52.2” E074º21’24.9” |
|  | Adamkay Cheema | N32º23’40.7” E074º21’38.8” |
|  | Bophal wala | N32º25’03.9” E074º21’47.0” |
|  | Sambrial | N32º29’05.6” E074º22’41.0” |
|  | Sambrial | N32º29’57.7” E074º22’39.9” |
|  | Airport Road | N32º31’35.3” E074º22’16.8” |
|  | Abiyala | N32º32’28.7” E074º23’32.0” |
|  | Kulluwal | N32º34’15.5” E074º27’01.8” |
|  | Khanna | N32º34’31.3” E074º27’44.6” |
|  | Khanna | N32º34’34.6” E074º27’54.8” |
|  | Khanna | N32º34’49.7” E074º28’23.8” |
|  | Bhuluwal | N32º34’37.2” E074º29’54.4” |
|  | Karimi choak | N32º36’39.7” E074º29’18.2” |
|  | Khajooriwal | N32º34’26.9” E074º29’46.4” |
|  | Peer Sabaz | N32º37’15.8” E074º27’49.8” |
|  | Darya Badar | N32º37’12.0” E074º27’24.1” |
|  | Bahadar Pur | N32º37’43.6” E074º26’55.5” |
|  | Head Marala Madina Town | N32º38’49.3” E074º28’23.8” |
|  | Machi Khokhar | N32º32’10.6” E074º30’12.2” |
|  | Behlol | N32º33’27.2” E074º30’22.4” |
|  | Laal pur | N32º33’24.6” E074º29’34.2” |
|  | Sandhu wala | N32º33’24.3” E074º29’13.6” |
|  | Kot Bura | N32º34’01.7” E074º28’18.3” |
|  | Dhagrai | N32º33’51.0” E074º27’50.3” |
|  | Nand Pur | N32º33’58.2” E074º28’29.2” |
|  | Bhula | N32º34’16.4” E074º30’02.5” |
|  | Bun | N32º29'34.6" E074º36'53.0" |

**Table S2.** Mean disease incidence and disease severity of bacterial stalk rot of maize recorded from different surveyed locations in district Sialkot, Pakistan.

| **Sr. No** | **Village/Area Name** | **Mean Disease Incidence%** | **Disease Severity (DS)** |
| --- | --- | --- | --- |
| 1. | Shahzada | 31.7 ± 4.8 | 2 |
| 2. | Kotli | 36.7 ± 1.7 | 2 |
| 3. | Kapoor pur | 17.7 ± 1.4 | 1 |
| 4. | Kamal pur | 61 ± 0.8 | 3 |
| 5. | Nawa pind | 67.5 ± 2.04 | 3 |
| 6. | Chota Shahzada | 21.5 ± 2.1 | 1 |
| 7. | Waain | 31 ± 1.4 | 2 |
| 8. | Chak raja | 32.5 ± 2.3 | 2 |
| 9. | Dugri | 12.5 ± 1.4 | 1 |
| 10. | Essa | 32.5 ± 3.4 | 2 |
| 11. | Chaiye wali | 31 ± 2.4 | 2 |
| 12. | Chuhan | 11 ± 1.7 | 1 |
| 13. | Pasrur | 20 ± 1 | 1 |
| 14. | Dhoal | 11 ± 0.8 | 1 |
| 15. | Kotli sayeda | 18.5 ± 1.7 | 1 |
| 16. | Rajiyan | 38.5 ± 2.7 | 2 |
| 17. | Nawa pind | 11 ± 2.0 | 1 |
| 18. | Chawinda | 15 ± 2.9 | 1 |
| 19. | Jasuran | 46.5 ± 2.10 | 3 |
| 20. | Sohedriky | 44 ± 3.7 | 3 |
| 21. | Badhyana | 48 ± 2.5 | 3 |
| 22. | Saho chuk | 12 ± 1.7 | 1 |
| 23. | Badi pur | 31 ± 1.7 | 2 |
| 24. | Nano wali | 19 ± 2.7 | 1 |
| 25. | Guri malihan | 38 ± 2.6 | 2 |
| 26. | Warion | 56 ± 2.0 | 3 |
| 27. | Bhutar | 57.4 ± 2.3 | 3 |
| 28. | Purab | 18.5 ± 1.7 | 1 |
| 29. | Bhati | 53.5 ± 2.08 | 3 |
| 30. | Nakhy | 48.5 ± 2.3 | 3 |
| 31. | Langy | 31 ± 2.08 | 2 |
| 32. | Manjhky | 35 ± 2.9 | 2 |
| 33. | Mali pur | 12.5 ± 2.08 | 1 |
| 34. | Nokrian | 47.5 ± 2.6 | 3 |
| 35. | Handle pura | 22.5 ± 1.4 | 1 |
| 36. | Kubay chak | 50 ± 5.9 | 3 |
| 37. | Pindi panjora | 15 ± 2.9 | 1 |
| 38. | Rum Adda | 35.5 ± 3.2 | 2 |
| 39. | Seedra khurd | 55 ± 2.8 | 3 |
| 40. | Khojay chak | 57.5 ± 2.3 | 3 |
| 41. | Bajwat | 43.5 ± 4.7 | 3 |
| 42. | Ugoke | 27 ± 2.9 | 2 |
| 43. | Wazirabad road | 28.5 ± 2.0 | 2 |
| 44. | Sialkot Airport Road | 37.4 ± 3.3 | 3 |
| 45. | Chak jeetea | 11 ± 1.4 | 1 |
| 46. | Abiala | 40 ± 6.0 | 3 |
| 47. | Mareeq pura | 27.5 ± 2.3 | 2 |
| 48. | Karnanwali | 15 ± 3.0 | 1 |
| 49. | Rao ghun | 32.5 ± 2.3 | 2 |
| 50. | Jugat pur | 12.5 ± 2.8 | 1 |
| 51. | Firdoos pura | 56 ± 3.2 | 3 |
| 52. | Kot dhara daa | 12 ± 2.3 | 1 |
| 53. | Nunaan wali kotli | 67.5 ± 3.4 | 4 |
| 54. | Bhoopal wala | 78.5 ± 4.5 | 4 |
| 55. | Wasarki adda | 66 ± 2.3 | 4 |
| 56. | Warsalke | 67.5 ± 2.3 | 4 |
| 57. | Adamky cheema | 25 ± 2.3 | 2 |
| 58. | Wasan kay | 13.5 ± 4.0 | 1 |
| 59. | Daska | 27 ± 2.9 | 2 |
| 60. | Deeto wali | 67.5 ± 2.6 | 4 |
| 61. | Channi gondal | 52 ± 1.5 | 3 |
| 62. | Deen pura | 42.5 ± 2.0 | 3 |
| 63. | Bhakhrewali | 68.5 ± 2.7 | 4 |
| 64. | Motra Bdyana road | 32.5 ± 1.5 | 2 |
| 65. | Dulam Ghazzi | 35 ± 4.4 | 2 |
| 66. | Bhagowaal | 52.5 ± 1.4 | 3 |
| 67. | Bakarpur | 37.5 ± 1.4 | 3 |
| 68. | Randhawa | 67.5 ± 2.0 | 4 |
| 69. | Kotli kurlan | 32.5 ± 2.0 | 2 |
| 70. | Miani | 42.5 ± 2.6 | 3 |
| 71. | Dilawarpur | 78 ± 2.0 | 4 |
| 72. | Khojay chak | 47.5 ± 2.8 | 3 |
| 73. | Najwal | 35 ± 2.9 | 2 |
| 74. | Chakpondra | 31 ± 1.4 | 2 |
| 75. | Sadra | 37.5 ± 1.4 | 2 |
| 76. | Chand Bagh | 23.5 ± 2.1 | 1 |
| 77. | Kapoor wali | 21.7 ± 2.8 | 1 |
| 78. | Kala kumra | 18 ± 2.3 | 1 |
| 79. | Sandhu wala | 41.5 ± 3.4 | 2 |
| 80. | Laal pur | 47.5 ± 1.5 | 2 |
| 81. | Shah Block | 32.5 ± 1.4 | 2 |
| 82. | Khumbranwala | 27.5 ± 3.1 | 2 |
| 83. | Abiyala | 31 ± 1.4 | 2 |
| 84. | Tanbray Basir | 66 ± 2.3 | 4 |
| 85. | Suraj | 74 ± 3.2 | 4 |
| 86. | Bangla Chowk | 76 ± 2.3 | 4 |
| 87. | Urah Chowk | 47.5 ± 1.4 | 3 |
| 88. | Syedan wali | 27.5 ± 1.4 | 2 |
| 89. | Raey pur | 13 ± 1.8 | 1 |
| 90. | Gopal pur | 11 ± 1.5 | 1 |
| 91. | Pathan wali | 32.5 ± 1.6 | 2 |
| 92. | Ledhoki | 15 ± 3.0 | 1 |
| 93. | Rang pur | 11 ± 1.4 | 1 |
| 94. | Dhuli | 14.5 ± 2.6 | 1 |
| 95. | Malany | 22.5 ± 2.0 | 1 |
| 96. | Plura | 23.5 ± 3.4 | 1 |
| 97. | Romal Jattan | 12 ± 2.0 | 1 |
| 98. | Kobay Chak | 46 ± 2.3 | 2 |
| 99. | Kammanwala | 23 ± 2.1 | 1 |
| 100. | Malhappar | 17.5 ± 1.5 | 1 |
| 101. | Kajlial | 37.5 ± 1.4 | 2 |
| 102. | Malagarpur | 27.5 ± 1.4 | 2 |
| 103. | Jhun | 37.5 ± 1.5 | 2 |
| 104. | Change | 42.5 ± 2.6 | 3 |
| 105. | Doburji Mallian | 12.5 ± 1.4 | 1 |
| 106. | Butter | 17.5 ± 3.1 | 1 |
| 107. | Pakki Kotli | 26 ± 2.3 | 2 |
| 108. | Miani | 32.5 ± 1.4 | 2 |
| 109. | Sadra Badra | 9.5 ± 1.2 | 1 |
| 110. | Addha | 27.5 ± 2.3 | 2 |
| 111. | Beera | 57.5 ± 2.3 | 3 |
| 112. | Hapu garaha | 48 ± 1.1 | 2 |
| 113. | Ghuinke | 20 ± 1.3 | 1 |
| 114. | Kotli Bagho | 32 ± 1.2 | 2 |
| 115. | Dhand pur | 63 ± 3.5 | 4 |
| 116. | Khrolian | 67.5 ± 1.8 | 4 |
| 117. | Raliyo kay | 63.5 ± 2.9 | 4 |
| 118. | Sahib kay | 37.5 ± 2.3 | 2 |
| 119. | Badho kay Cheema | 67.5 ± 2.0 | 4 |
| 120. | Mandyianwala | 11 ± 1.4 | 1 |
| 121. | Walany | 28.5 ± 1.4 | 2 |
| 122. | Satowali | 11.5 ± 1.7 | 1 |
| 123. | Chouni Slehrian | 47.5 ± 1.4 | 3 |
| 124. | Pico shore | 19.5 ± 0.8 | 1 |
| 125. | Kalan Joudhay wali | 18.5 ± 1.4 | 1 |
| 126. | Ghalotian Road | 19.3 ± 3.3 | 1 |
| 127. | Musalmanian | 26 ± 3.3 | 1 |
| 128. | Othian | 18.5 ± 1.7 | 1 |
| 129. | Boobkan wala | 8.5 ± 1.4 | 1 |
| 130. | Gujranwala Road | 22.5 ± 1.5 | 2 |
| 131. | Mandrawala | 30 ± 1.3 | 2 |
| 132. | Bophal wala | 66.5 ± 1.2 | 4 |
| 133. | Sambrial | 24.5 ± 3.2 | 2 |
| 134. | Kulluwal | 46.5 ± 2.0 | 3 |
| 135. | Khanna | 18 ± 1.3 | 1 |
| 136. | Bhuluwal | 18.5 ± 1.7 | 1 |
| 137. | Karimi choak | 31 ± 1.7 | 2 |
| 138. | Khajooriwal | 48.5 ± 2.3 | 3 |
| 139. | Peer Sabaz | 68.5 ± 2.0 | 4 |
| 140. | Darya Badar | 21.5 ± 2.0 | 2 |
| 141. | Bahadr pur | 39 ± 1.7 | 2 |
| 142. | Head Marala Madina Town | 9 ± 1.1 | 1 |
| 143. | Machi Khokhar | 11.5 ± 0.8 | 1 |
| 144. | Behlol | 61 ± 0.8 | 4 |
| 145. | Laal pur | 57.5 ± 1.5 | 3 |
| 146. | Sandhu wala | 73 ± 2.3 | 4 |
| 147. | Kot Bura | 47.5 ± 1.4 | 3 |
| 148. | Dhagrai | 67.5 ± 1.4 | 4 |
| 149. | Nand Pur | 71.5 ± 0.8 | 4 |
| 150. | Bhula | 67.5 ± 1.4 | 4 |
| 151. | Bun | 11 ± 1.4 | 1 |
